# Editorial: The Biological Landscape of Immunotherapy in AML

**DOI:** 10.3389/fonc.2021.671252

**Published:** 2021-04-15

**Authors:** Alessandro Isidori, Naval Daver, Antonio Curti

**Affiliations:** ^1^ Hematology and Stem Cell Transplant Center, Azienda Ospedaliera Ospedali Riuniti Marche Nord (AORMN) Hospital, Pesaro, Italy; ^2^ Department of Leukemia, MD Anderson Cancer Center, Houston, TX, United States; ^3^ Istituto di Ematologia Seràgnoli, IRCCS Azienda Ospedaliero-Universitaria di Bologna, Bologna, Italy

**Keywords:** acute myeloid leukemia, immunotherapy, cell biology, immune escape, immunoediting of cancer

##  

Acute myeloid leukemia (AML) is a molecularly and clinically heterogeneous hematologic malignancy that progresses rapidly and originates from a rare population of leukemic stem cells. Despite intensive chemotherapy followed by allogeneic stem cell transplantation and the recent approval of new targeted agents, the overall outcome of AML is still unsatisfactory, with an estimated 5-year overall survival (OS) of approximately 30% (1). In this scenario, investigation of innovative strategies and tools, such as immunotherapies, is highly warranted to improve the overall clinical outcome of patients with AML.

In the last 5-10 years, knowledge regarding the role of the immune microenvironment in the pathophysiology of AML has increased significantly. A growing body of evidence indicates that, similar to most malignancies, the functional interplay between leukemic cells and bone marrow (BM) immune microenvironment comprises a distinctive hallmark of AML. AML cells interact with a variety of immune cells, which crucially influence proliferation, survival, and drug resistance, by creating an immunosuppressive microenvironment (2). Both innate and adaptive immune responses are involved and profoundly dysregulated in the presence of AML in the bone marrow, thus leading to tolerance induction and leukemia escape from immune control (3). The understanding of the mechanisms involved in the interaction between AML and immune cells within the BM microenvironment, and the identification of factors responsible for the escape of leukemic cells from immune surveillance, are crucial steps toward the development and application of innovative immunotherapeutic approaches and therapies. The major aim of these therapies is to harness the immune system against AML, both by eliciting the cytotoxic effector pathways, such as cytotoxic T lymphocytes (CTLs) and natural killer (NK) cells, and/or by inhibiting the tolerogenic mechanisms, such as T regulatory cells (Tregs), suppressive macrophages and mesenchymal stromal cells (MSCs). In this Research Topic, we discuss some recent advances in understanding these processes, focusing on the biologic landscape of AML immunotherapy.

Mougiakakos et al. provide a comprehensive review of the metabolic changes occurring in AML cells that lead to immune-escape. Noting that one of the cancer hallmarks is metabolic reprogramming and that metabolic adaptation is crucial for tumor development and progression, the authors discuss recent evidence indicating the role of alterations in cell metabolism for immune escape. Metabolic alterations are linked to immune-regulation and to a deep metabolic re-modeling of the microenvironment that favors an immune-suppressive phenotype. In particular, the induction of immune-regulatory cell subsets, such as regulatory T cells (Tregs) is highlighted. A correlation of these metabolic changes with oncogenic mutations is outlined and discussed by providing a comprehensive picture of potentially suitable countermeasures and their limitations.

Ciciarello et al. focus on the emerging mechanisms deriving from the BM microenvironment and conferring a survival advantage to AML cells over normal hematopoietic stem cells (HSCs). In particular, the authors tackle the role of MSCs, which have, for a long time, been recognized as pivotal contributors in the setup and maintenance of the HSC niche. More recently, MSCs have emerged as crucial regulators of the immune response, mainly toward the induction of immune tolerance. Indeed, it has been demonstrated that MSCs create a protective and immune-tolerant microenvironment, which supports the survival of leukemic cells and limits response to therapies. Finally, it provides a comprehensive overview of the emerging MSC-driven mechanisms of drug resistance, including the effects of soluble factors versus cell-to-cell contact, the regulation of redox homeostasis as well as of mitochondrial metabolism, and the role of inflammatory signaling.

In their review, Ocadlikova et al. summarize current knowledge about the mechanisms and effects by which chemotherapy remodels the leukemia immune microenvironment. It is well known that some chemotherapeutic agents, such as anthracyclines, may activate an anti-tumor immune response in a process known as chemotherapy-induced immunogenic cell death. Nonetheless, emerging evidence indicates that tolerogenic mechanisms and immunosuppressive pathways may be concomitantly induced. In this regard, a specific focus is dedicated to dendritic cells (DCs) which, in the presence of leukemic blasts, may be driven to acquire tolerogenic features such as the overexpression of indoleamine 2,3-dioxygensase, thus expanding Tregs. A better understanding of the whole process underlying chemotherapy-induced alterations of the immunological tumor microenvironment have important clinical implications to fully leverage the immunogenic potential of anti-leukemia agents and fine tune their application. The combination of immunogenic chemotherapy with immunotherapies may represent an interesting area of preclinical and clinical investigation.

Although the recurrent genetic mutations found in AML have been intensively studied from a cell-intrinsic perspective, leading to the genesis of multiple, recently approved, targeted therapies, there is a paucity of data on the effects of these targeted agents on the leukemia microenvironment, including the immune system. In their work, Mendez et al. summarize the most recent advances in understanding the complex interplay between the genetic and immune landscapes of AML for novel risk stratification and therapeutic approaches. Based on the growing body of evidence that places the crosstalk with the immune system at the crux of any effective therapy, ongoing and future research will reveal how AML genetics informs and alters the composition of the immune microenvironment. After discussing the most recent advances in the understanding of the effects of anti-leukemia agents on the immune landscape, the authors present the recurrent genetic mutations found in AML from an innovative cell-extrinsic perspective. Although still poorly elucidated, this article discusses the mechanisms by which recurrent genetic mutations in AML, such as *NPM1, FLT3-ITD, IDH1/2*, and *TP53* can modulate the immune microenvironment in AML.

Sweeney et al. focus their review on the graft-versus-leukemia (GvL) effect in AML. Allogeneic hematopoietic stem cell transplantation (allo-SCT) is the most potent anti-leukemic therapy in patients with AML and one of the first examples of curative immunotherapy in oncology. Preclinical and clinical advances understanding the complex mechanisms underlying GvL and graft-versus-host (GvH) effects are discussed focusing on strategies to fully exploit the anti-leukemia immunological potential of allo-SCT (GvL) whilst minimizing the toxicity of GvH.

There is increasing evidence of the crucial role played by components of the innate immune system within the leukemia microenvironment. Chao et al. review the role of CD47, recently identified as a dominant macrophage inhibitory checkpoint, in myeloid malignancies and pre-clinical data supporting CD47 targeting. Initial clinical data of CD47 targeting in AML/MDS are reviewed, including the first-in-class anti-CD47 antibody, magrolimab, as a single agent and in combination with hypomethylating agents. Early clinical data highlight the importance of targeting immune checkpoints such as CD47 and the critical role for macrophages in the pathophysiology of leukemic disease. At the translational level, key questions that need to be addressed including biomarker strategies for patient selection and the identification of effective therapeutic combinations with CD47 blockade, are discussed.

In summary, the collection of review articles in this Research Topic provide a timely update on the progress made in the understanding of the biologic landscape of immunotherapy in AML. Although major strides have been made, resulting in a deeper understanding of the mechanisms underlying the role of the immune microenvironment in the pathophysiology of AML, to fully exploit and translate immunotherapy into a clinical effective modality a long list of unanswered questions remains to be answered, from the biologic perspective. Among them, the question of whether immune biomarkers capture the biological significance of the microenvironment in AML biology and response to therapy represents a major task with important implications. In many cancers, a variety of immune-specific gene expression patterns, which profile the immune microenvironment, have been recently described as predictive of the outcome in patients treated with conventional chemotherapy and, more importantly, with novel immunotherapy agents (4–9). In AML, the current risk-assessment classification^1^ and prognostication are still predominantly focused on cytogenetic and molecular alterations, which are historically known to influence the sensitivity of leukemic cells to conventional chemotherapy. However, based on the compelling preclinical data regarding the fundamental impact of tolerogenic tumor microenvironment mechanisms in dysregulating patients’ immune response, the prognostic significance of the immune landscape is recognized as being increasingly important in AML, revealing that immune-related genes may predict survival (10). Based on these premises, it is time to move in the direction of a paradigm shift with the ambition to refine stratification and prognostication of AML patients that incorporates an immunological-driven biological approaches. In this scenario, panels of immune-based biomarkers, predictive of response to immune therapy, need to be tested in prospective clinical trials.

In conclusion, to fully enter into the immunotherapy era and develop novel immunotherapy drugs and strategies, we need a comprehensive and continually deepening understanding of the immune-biological landscape of AML.

## Author Contributions

All authors equally wrote and contributed to the manuscript. All authors contributed to the article and approved the submitted version.

## Conflict of Interest

The authors declare that the research was conducted in the absence of any commercial or financial relationships that could be construed as a potential conflict of interest.
